# Supplementary material for: A Global Screen for Assembly State Changes of the Mitotic Proteome by SEC-SWATH-MS
Source: Cell Syst. 2020 Feb 26;10(2):133–155.e6. doi: 10.1016/j.cels.2020.01.001 (PMC7042714; doi:10.1016/j.cels.2020.01.001)

Q8N4C8 | MINK1_HUMAN | MINK1 B55 MAP4K6 MINK YSK2 ZC3
Monomer MW [kDa]: 149.822 Monomer expected elution fraction: 37

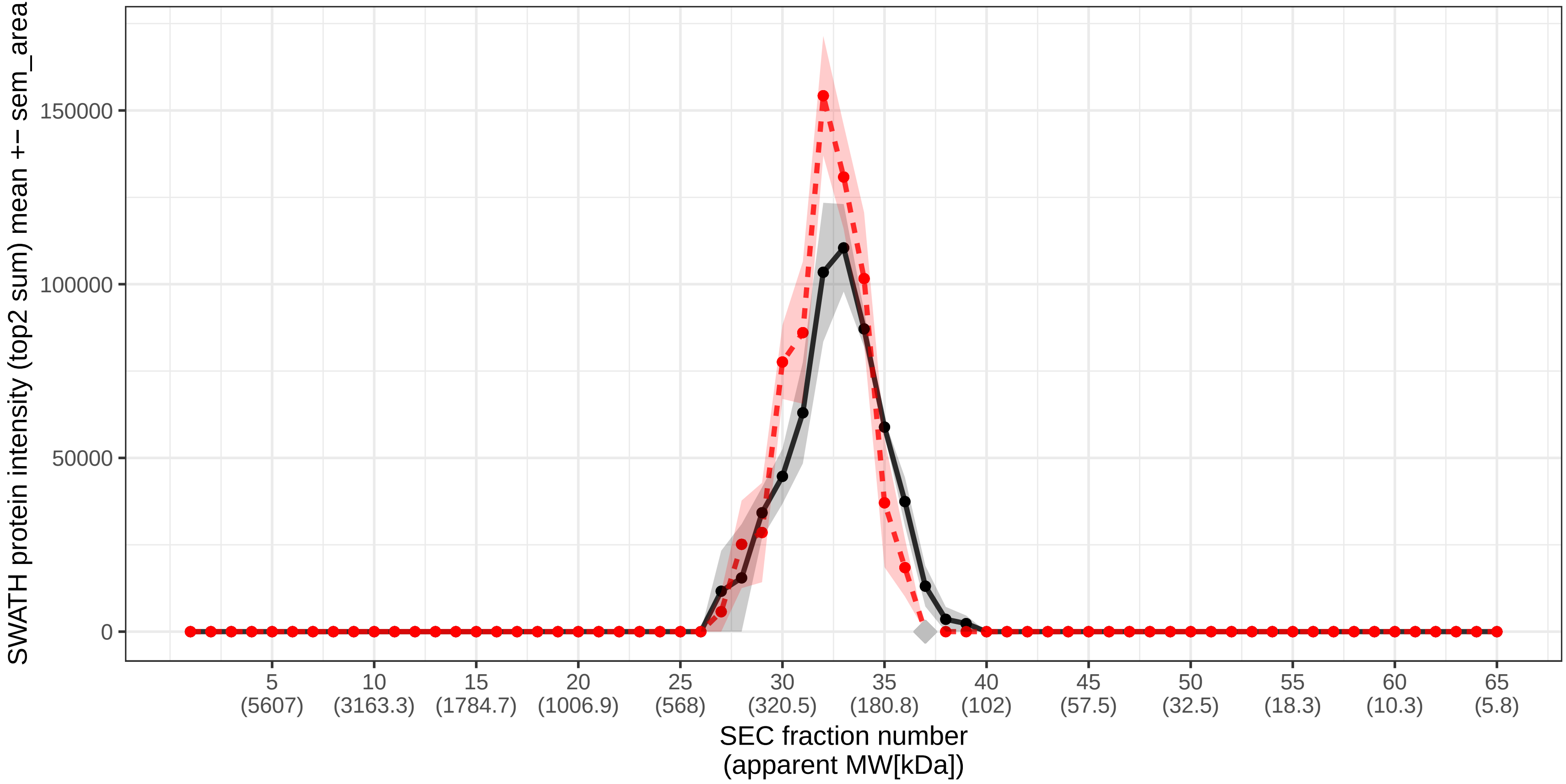

Supplement: Data S1. SEC-SWATH-MS Protein Chromatograms, Related to Figure 1 [file mmc6.zip › SECchrom_Q8N4C8_MINK1_HUMAN_MINK1_B55_MAP4K6_MINK_YSK2_ZC3.pdf]
